# Surface Active to Non-Surface Active Transition and Micellization Behaviour of Zwitterionic Amphiphilic Diblock Copolymers: Hydrophobicity and Salt Dependency

**DOI:** 10.3390/polym9090412

**Published:** 2017-09-05

**Authors:** Sivanantham Murugaboopathy, Hideki Matsuoka

**Affiliations:** 1 Department of Polymer Chemistry, Kyoto University, Katsura, Kyoto 615-8510, Japan; sivanantham@prist.ac.in; 2 Centre for Research and Development, Department of Physics, PRIST University, Vallam, Thanjavur 613 403, Tamil Nadu, India

**Keywords:** non-surface activity, self-assembly, hydrophobicity, zwitterionic amphiphilic block copolymer, light scattering, polymer micelle

## Abstract

We have synthesized a range of zwitterionic amphiphilic diblock copolymers with the same hydrophilic block (carboxybetaine) but with different hydrophobic blocks (*n*-butylmethacrylate (*n*-BMA) or 2-ethylhexylacrylate (EHA)) by the reversible addition–fragmentation chain transfer (RAFT) polymerization method. Herein, we systematically examined the role of hydrophobicity and salt concentration dependency of surface activity and micellization behaviour of block copolymer. Transition from surface active to non-surface active occurred with increasing hydrophobicity of the hydrophobic block of block copolymer (i.e., replacing P(*n*-BMA) by PEHA). Foam formation of block copolymer slightly decreased with the similar variation of the hydrophobic block of block copolymer. Block copolymer with higher hydrophobicity preferred micelle formation rather than adsorption at the air–water interface. Dynamic light scattering studies showed that block copolymer having P(*n*-BMA) produced near-monodisperse micelles, whereas block copolymer composed of PEHA produced polydisperse micelles. Zimm plot results revealed that the value of the second virial coefficient (*A*_2_) changed from positive to negative when the hydrophobic block of block copolymer was changed from P(*n*-BMA) to PEHA. This indicates that the solubility of block copolymer having P(*n*-BMA) in water may be higher than that of block copolymer having PEHA in water. Unlike ionic amphiphilic block copolymer micelles, the micellar shape of zwitterionic amphiphilic block copolymer micelles is not affected by addition of salt, with a value of packing parameters of block copolymer micelles of less than 0.3.

## 1. Introduction

Over the past few decades, amphiphilic block copolymers are gaining attention because of their wide range of applications in our daily life, in either solid or solution form [1,2,3]. They have been used as thermoplastic elastomers, drug delivery systems, emulsifiers, coating materials and templating materials in nano-lithography [1,2,3,4]. Studies on novel amphiphilic block copolymers with novel properties are being conducted.

Amphiphilic block copolymers with various kinds of ionic groups such as anionic, cationic, non-ionic and zwitterionic have been studied by different research groups [5,6,7,8,9,10,11,12,13,14,15,16,17,18,19,20,21,22]. In particular, ionic amphiphilic block copolymer (IABC) systems have been studied extensively [5,6,7,8,9,10,11,12,13,14,15,16,17,18], whereas zwitterionic amphiphilic block copolymers (ZABCs) have hardly been studied [19,20,21,22]. In the IABC systems, several factors such as molecular weight of both hydrophobic and hydrophilic blocks of block copolymers, salt, pH, type of ionizing groups (strong or weak), glass transition temperature of hydrophobic block affect their properties namely, surface activity, foam formation and micellization behaviour [7,8,9,10,11,12,13,14,15]. Kaewsaiha et al. [10] reported that IABC showed non-surface activity when IABC had comparable hydrophilic and hydrophobic block lengths but surface activity was observed when the hydrophobic block length was three times longer than the hydrophilic block. In that study, hydrophobic adsorption force, which depends on the relative molecular weight of hydrophobic and hydrophilic blocks, played a vital role in deciding the surface activity of IABC. Matsuoka and his co-workers [14,15] have examined the role of molecular weight on surface activity and micellization behaviours of IABC. Molecular weight of the polymers decided the non-surface activity of IABC. The degree of polymerization must be more than 30 for both ionic and hydrophobic blocks to be non-surface active [14]. A longer ionic block length also suppresses non-surface activity [15]. The role of hydrophobicity on non-surface activity behaviours of IABC having the same hydrophilic block (poly(styrene sulphonate)) and different hydrophobic blocks ((poly(*n*-butyl acrylate), polystyrene, and poly(pentafluorostyrene)) has also been investigated [14]. IABC with higher hydrophobicity had higher non-surface activity. This phenomenon can be explained by stable micelle formation: a micelle situation with the highest hydrophobic IABC is more stable than the adsorped state at the air–water interface. Hence, stable micelle formation was found to be one of the key factors for non-surface activity. Another important factor is the image charge effect [14,15]. The image charge effect occurred because of the presence of polyions in the ionic block and these polyions were electrostatically repelled from the air–water interface by the image charge effect at the air–water interface [23]. In fact, transition from non-surface active to surface active was observed by salt addition [8,9,10]. Hence, two more vital conditions, i.e., the image charge effect and stable micelle formation, are key factors for the non-surface activity of IABC.

Theodoly et al. [18] recently reported that formation of frozen micelles is the main criteria for non-surface activity. However, we found that non-surface activity was exhibited by non-frozen micelles obtained from poly(hydrogenated isoprene)-*b*-poly(styrene sulphonate) [11]. In their study, the formation of the non-frozen micelle was confirmed by small-angle neutron scattering measurements that revealed the micellar structural transition from sphere to rod after salt addition.

Recently, we investigated the effect of salt on the surface activity and micellization behaviour of ZABC containing *n*-butylacrylate (*n*-BA) and carboxybetaine [22], and found that ZABC underwent transition from surface active to non-surface active by addition of salt. We also found that the surface activity and micellization behaviour of ZABC were opposite those of IABC both in the presence and absence of salt. In this study, we carried out systematic investigation on the role of hydrophobicity of the hydrophobic block on the surface activity and micellization behaviour of ZABC using block copolymer composed of *n*-butylmethacrylate (*n*-BMA) or 2-ethylhexylacrylate (EHA) as a hydrophobic block and carboxybetaine as a hydrophilic block. The hydrophobicity of EHA is higher than that of either *n*-BMA or *n*-BA (order of hydrophobicity: EHA > *n*-BMA > *n*-BA). [24,25]. In addition, the glass transition temperature of *n*-BMA (20 °C) is higher than that of either *n*-BA (−54 °C) or EHA (−70 °C) [26]. Therefore, we studied the effect of hydrophobicity, salt, block length and glass transition temperature on the surface activity and micellization behaviour of ZABC. Surface tension, static light scattering (SLS) and dynamic light scattering (DLS) measurements were applied to carefully and systematically investigate the interfacial properties of ZABC at air–water interface as well as their hydrodynamic properties in aqueous media.

## 2. Experimental Section

### 2.1. Materials

*n*-Butylmethacrylate (*n*-BMA), 2-ethylhexylacrylate (EHA), 4,4′-azocyanovaleric acid (ACVA), 2,2′-azobisisobutyronitrile (AIBN), *N*,*N*-dimethylformamide (DMF),diethyl ether and methanol were products of Wako Pure Chemicals (Osaka, Japan). Carboxybetaine (GLBT) was supplied by Osaka Organic Chemicals Co. Ltd. (Osaka, Japan). Distillation method was used to purify *n*-BMA and EHA. The chain transfer agent (CTA) 4-cyanopentanoic acid-4-dithiobenzoate was synthesized as reported previously [27,28]. Deuterium oxide (D_2_O), deuterated methanol (CD_3_OD) anddeuterated chloroform (CDCl_3_) were product of Cambridge Isotope Laboratory (Tewksbury, MA, USA). Water used for sample solution preparation and dialysis was ultrapure water of resistance 18 MΩ cm by the Milli-Q System (Millipore, Bedford, MA, USA).

### 2.2. Synthesis

**Scheme 1:**
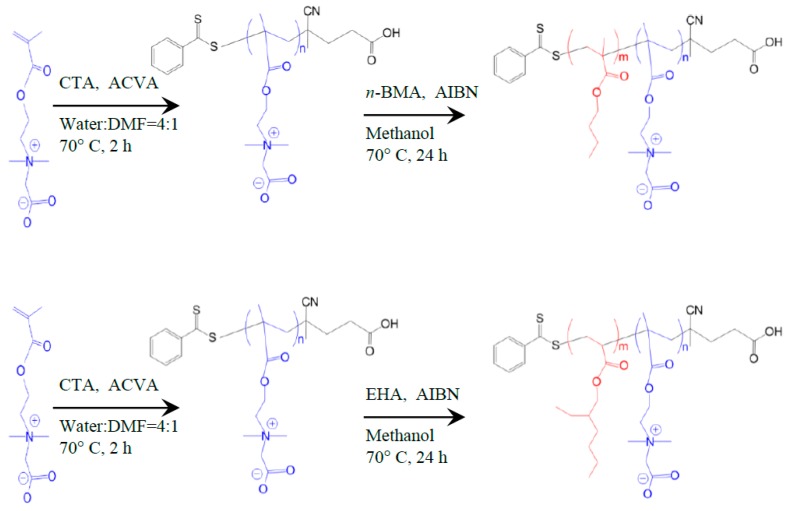
Synthesis of homopolymer (PGLBT) and diblock copolymers (P(*n*-BMA)-*b*-PGLBT) and (PEHA-*b*-PGLBT).

**Figure 1:**
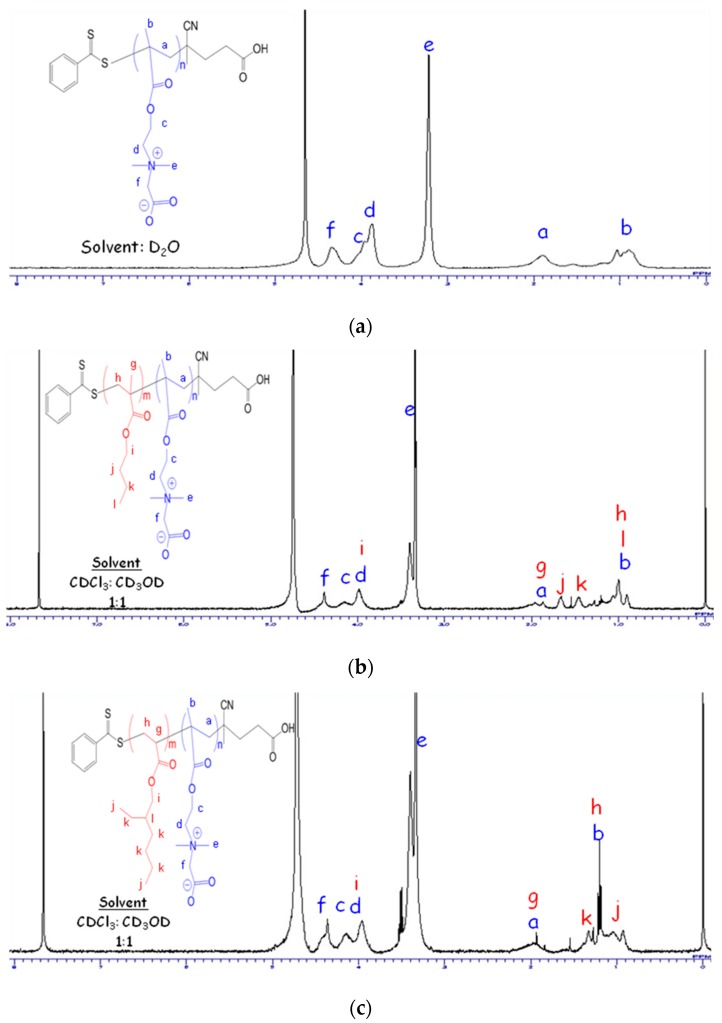
^1^H NMR spectra of: (**a**) homopolymer PGLBT in D_2_O; (**b**) diblock copolymer P(*n*-BMA)-*b*-PGLBT in CDCl_3_:CD_3_OD (1:1); and (**c**) diblock copolymer P(EHA)-*b*-PGLBT in CDCl_3_:CD_3_OD (1:1).

Scheme 1 shows the method of synthesis of both homopolymers (PGLBT) and diblock copolymers (P(*n*-BMA)-*b*-PGLBT) and (PEHA-*b*-PGLBT) with various block lengths via reversible addition–fragmentation polymerization (RAFT). The polymerization conditions used for the synthesis of homopolymers (PGLBT) are shown in Table S1. First, GLBT, CTA and ACVA were mixed well with the mixed solvents containing water and DMF at the ratioof 4:1 in a Schlenk tube using a magnetic stirrer. Then, the mixture was degassed under Ar gas atmosphere for three freeze–pump–thaw cycles and then filled with Ar gas. After degassed, RAFT polymerization was carried out at 70 °C for 2 h. When the polymerization was completed, the product was dialyzed in Milli-Q water for 3 days and the homopolymer PGLBT was extracted by freeze-drying. GPC experiments were conducted to determine the molecular weight and its distribution of PGLBT and results are summarized in Table S2. ^1^H NMR experiments (see Figure 1) were used to confirm the formation of homopolymers (PGLBT).

### 2.3. ^1^H Nuclear Magnetic Resonance (^1^H NMR)

^1^H NMR spectra of homopolymer and block copolymers were recorded using a JEOL 400WS (JEOL, Tokyo, Japan). To record the ^1^H NMR spectra of homopolymers (macro CTA-PGLBT), we used D_2_O as a solvent. However, mixed solvents ofCD_3_OD and CDCl_3_ at a ratio of 1:1 were used for recording the ^1^H NMR spectra of diblock copolymers (P(*n*-BMA)-*b*-PGLBT) and (PEHA-*b*-PGLBT).

### 2.4. Gel Permeation Chromatography (GPC)

GPC measurements were conducted using a JASCO system (Tokyo, Japan) LC-2000 with a UV detector (UV-2075), a refractive index detector (RI-2031) and a Shodex OH pack (SB-804 HQ).The eluent was a mixture of 0.3 M sodium sulphate (Na_2_SO_4_) and 0.5 M acetic acid (CH_3_COOH) adjusted topH3. The sample solution of concentration of 2 mg/mL was used for injection. The number averaged molecular weight (*M*_n_) and the polydispersity index (*M*_w_/*M*_n_) of PGLBT (macro CTA) are shown in Table S2.

### 2.5. Surface Tension Measurements

Surface tension of polymer solutions was measured by a FACE CBVP-Z Surface Tensiometer (Kyowa Interface Science Co., Ltd., Tokyo, Japan) by the Wilhelmy plate (platinum) method.

### 2.6. Foam Formation and Foam Height Measurements

Block copolymer solutions of concentration of 1 mg/mL were prepared using Milli-Q water with or without salt (1 M NaCl). These solutions were mechanically shaken for 1 min in identical containers to check the foam forming ability of these polymer solutions. Foam height was also measured as a function of time.

### 2.7. Light Scattering Measurements

Photal DLS-7000 light scattering setup (Otsuka Electronic, Osaka, Japan) was used for SLS and DLS measurements. This setup was composed of a goniometer, a multi-tau correlator (GC-1000) and a 15 mW He–Ne laser with a wavelength of 632.8 nm. The intensity–intensity autocorrelation function (ICF) $g_{}^{\left(2\right)}\left(q,t\right)$ was measured at different scattering vectors q=(4πn/λ)sin(θ/2), where *n* is the refractive index of the solvent, *θ* is the scattering angle and *λ* is the wavelength of incident laser beam. Using Siegert relation [29], the field correlation function $g_{}^{\left(1\right)}\left(q,t\right)$ was obtained from ICF $g_{}^{\left(2\right)}\left(q,t\right)$. From the field correlation function $g_{}^{\left(1\right)}\left(q,t\right)$, the decay rate *Г* was evaluated by single or double-exponential fitting. From the slope of the plot of *Г* vs. *q*^2^, the translational diffusion coefficient (*D*) was calculated using the relation ($\Gamma =Dq^{2}$). The Stokes-Einstein equation was used to determine the hydrodynamic radius (*R*_h_) from the value of *D*.

SLS measurements were carried out by varying the scattering angle (*θ*) from 30° to 150° at 10° intervals. Zimm plots were used to obtain the weight-average molecular weight (*M*_w_), radius of gyration (*R*_g_) and second virial coefficient (*A*_2_) using the following equation [30,31].

(1) $$\frac{KC_{p}}{I\left(q\right)}=\frac{1}{M_{w}}\left(1+\frac{1}{3}{\langle R_{g}\rangle }^{2}q^{2}\right)+2A_{2}C_{p}$$

where *K* is an optical constant ($4\pi^{2}n^{2}{\left(dn/dC_{p}\right)}^{2}/N_{A}\lambda^{4}$), *I(q)* is the scattered intensity at given *q*, *C*_p_ is the polymer concentration, *dn/dC_p_* is the refractive index increment against *C_p_*, and *N*_A_ is Avogadro’s number.

### 2.8. Specific Refractive Index Increment Measurements (dn/dc_p_)

Photal differential refractometer DRM-3000S (Otsuka Electronic, Osaka, Japan) was used to determine the specific refractive index increment (*dn*/*dc_p_*).This instrument had a He-Ne laser (wavelength: 632.8 nm) as a light source.

## 3. Results and Discussion

### 3.1. Hydrophobicity and Salt-Dependent Air–Water Interfacial Properties

Previously, we investigated the influence of salt on the surface activity and micellization behaviour of ZABC containing *n*-BA and carboxybetaine [22]. In that study, ZABC having *n*-BA showed transition from surface active to non-surface active after addition of salt. The surface activity behaviour of ZABC was opposite that of IABC both in the presence and absence of salt. Herein, we examined the role of hydrophobicity and salt on the surface activity and micellization behaviour of ZABC having *n*-BMA or EHA as a hydrophobic block and carboxybetaine as a hydrophilic block. As mentioned earlier, the hydrophobicity of EHA is higher than that of *n*-BMA and *n*-BA (order of hydrophobicity: EHA> *n*-BMA > *n*-BA) [24]. Thus, we examined how the hydrophobicity of hydrophobic block of ZABC affects their air–water interfacial properties.

**Figure 2:**
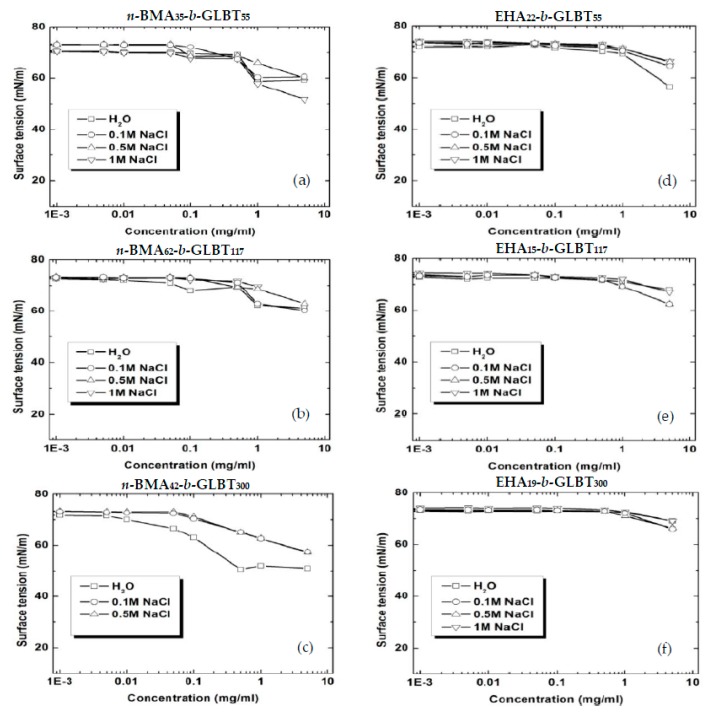
Comparison of the surface tension of ZABC’s having P(*n*-BMA) and PEHA as a function of their concentrations in water and various concentrations of sodium chloride solutions.

To explore the influence of hydrophobicity and salt on the surface tension of different ZABCs, we measured the surface tension on ZABCs with P(*n*-BMA) and PEHA and variation of the surface tension is plotted as a function of polymer concentrations, as shown in Figure 2. First, we describe the effect of hydrophobicity on the surface activity of ZABC. ZABC having P(*n*-BMA) showed moderate surface active behaviour at higher polymer concentrations (predominant for *n*-BMA_42_-*b*-GLBT_300_) (Figure 2a–c). This indicates that ZABC with P(*n*-BMA) in water behaves like an almost surface active polymer which means that ZABC may be adsorped at the air–water interface (higher concentration region). This phenomenon was analogous to that of ZABC with P(*n*-BA) [22]. ZABC having P(*n*-BA) was more surface active than ZABC having P(*n*-BMA) [22]. This could be attributed to the lower hydrophobicity of the hydrophobic block P(*n*-BA) compared with that of P(*n*-BMA). Armes et al. [19] reported that the block copolymers having sulphopropylbetaine was slightly surface active. In contrast, almost non-surface active nature was observed when the P(*n*-BMA) block of ZABC was replaced by PEHA (Figure 2d–f). This implies that ZABC having a higher hydrophobic block (PEHA) acts like a non-surface active polymer which prefers a micelle state rather than adsorped state at the air–water interface since the former is more stable than latter. Earlier, Matsuoka et al. [14] reported the effect of hydrophobicity on non-surface activity behaviours of IABC having the same hydrophilic block (poly-(styrene sulphonate)) and different hydrophobic blocks ((poly(*n*-butyl acrylate), polystyrene, and poly(pentafluorostyrene)). The more hydrophobic is the hydrophobic block of IABC, the higher is its non-surface activity. Hence, our observations on the non-surface activity of ZABC are consistent with the previous studies on non-surface activity of IABC [14,15]. Hence, a stable micelle formation is one of the key factors of the non-surface active nature also for ZABC in addition to IABC.

Next, we examined the role of salt addition on the surface activity of ZABC. The surface tension of ZABC containing P(*n*-BMA) (specifically for *n*-BMA_42_-*b*-GLBT_300_) with increase in polymer concentration seems to decrease after addition of salt (Figure 2c). This implies that, upon addition of salt, the ZABC with P(*n*-BMA) (mainly, *n*-BMA_42_-*b*-GLBT_300_) undergoes transition from slightly surface active to non-surface active. Recently similar transition was observed for ZABC with P(*n*-BA) [22]. This transition was attributed to conversion of betaine (zwitterionic) into anionic polymer in the presence of salt which was reported previously [32,33,34,35,36]. This might be related to the change of situation of betaine ions: They are in intra- and intermolecular salts in the absence of added salt, but they change to ions with counterions with added salt ions. On the other hand, salt addition increases the adsorption of IABC at the air–water interface considerably [7,8,9,10,11,12,13,14]. This is due to the screening of image charge effect by added salt ions. Hence, the salt-dependent surface tension behaviour of ZABC showed a trend opposite that of IABC reported previously [14,15,18]. However, salt addition hardly affected the surface activity of ZABC containing PEHA (Figure 2d–f).

**Figure 3:**
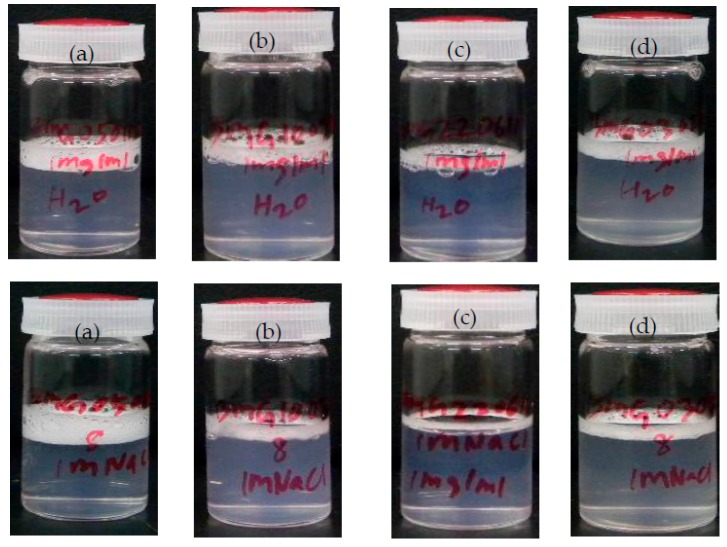
Influence of chain length and salt on foam forming abilities of different ZABCs containing P(*n*-BMA): (**a**) *n*-BMA_35_-*b*-GLBT_55_; (**b**) *n*-BMA_62_-*b*-GLBT_117_; (**c**) *n*-BMA_101_-*b*-GLBT_156_; and (**d**) *n*-BMA_42_-*b*-GLBT_300_ of concentration (1 mg/mL) in water (**top row**) and 1 MNaCl (**bottom row**).

**Figure 4:**
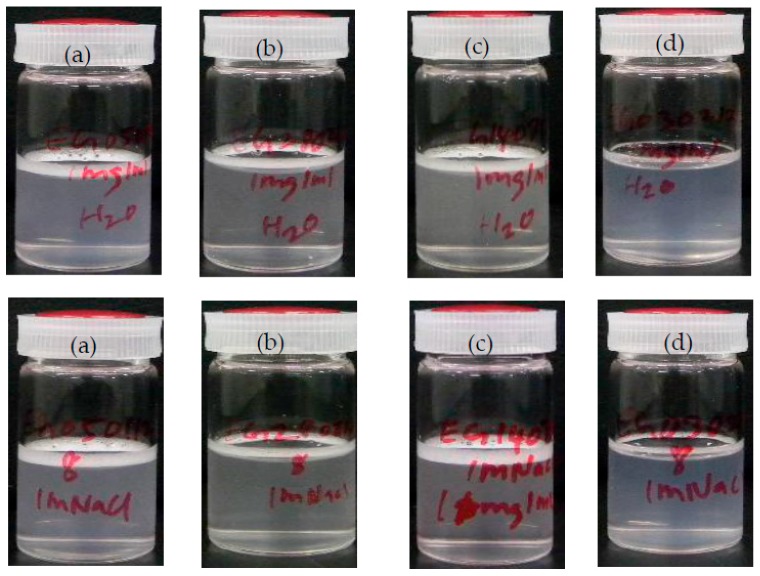
Effect of chain length and salt on foam forming abilities of various ZABCs having PEHA: (**a**) EHA_22_-*b*-GLBT_55_; (**b**) EHA_15_-*b*-GLBT_117_; (**c**) EHA_20_-*b*-GLBT_156_; and (**d**) EHA_19_-*b*-GLBT_300_ of concentration (1 mg/mL) in water (**top row**) and 1 M NaCl (**bottom row**).

The effects of hydrophobicity, salt and block length on the foam forming behaviour of ZABC have also been investigated. Figure 3 and Figure 4 show the hydrophobicity and chain length-dependent foam-forming behaviour of ZABC solutions in the absence and the presence of 1 M sodium chloride, respectively. These figures clearly show that the net hydrophobicity and chain length of ZABC are the dominant factors for the foam forming behaviour of ZABC. The higher are the net hydrophobicity and block length of the block copolymer, the lower is the foam formation. For instance, since ZABC having PEHA is more hydrophobic than those having P(*n*-BMA), the former showed poorer foam forming behaviour than the latter. The foam forming behaviour of ZABC with P(*n*-BMA), decreased with the increase in block length except for *n*-BMA_42_-*b*-GLBT_300_. The reason for this behaviour could be the smaller net hydrophobicity of *n*-BMA_42_-*b*-GLBT_300_ than *n*-BMA_101_-*b*-GLBT_156_. We observed a similar trend of decrease in foam formation with the increase in block length for ZABC with P(*n*-BA) [22]. This might be due to a larger amount of adsorbed polymer with lower molecular weight at the air–water interface than that with higher molecular weight polymers. This might be related to micelle formation in bulk and its stability. Foam formation can be related to CMC of polymers that were determined from SLS measurements. CMC of the polymers such as EHA_22_-*b*-GLBT_55_, EHA_15_-*b*-GLBT_117_ and EHA_20_-*b*-GLBT_156_, respectively, were 0.006, 0.01 and 0.03 mg/mL in water, whereas the CMC of these polymers in 1 M NaCl were 0.0032, 0.0034 and 0.007 mg/mL. Smaller values of CMCs indicate that polymers prefer stable micelle formation even at lower concentration of polymers rather than adsorped at air–water interface. Some of the polymer solutions, particularly ZABC with P(*n*-BMA) except *n*-BMA_42_-*b*-GLBT_300_, appear bluish, which implies that these solutions have near monodisperse micelles. In the next section, we will explain the evidence for the presence of near-monodisperse micelles in terms of polydispersity indices of micelles. A higher balance/symmetry between hydrophobic and hydrophilic blocks may be the key factor for the bluishness of these micellar solutions. For example, polymer solutions that are formed from the block copolymers *n*-BMA_35_-*b*-GLBT_55_, *n*-BMA_62_-*b*-GLBT_117_ and *n*-BMA_101_-*b*-GLBT_156_ appear bluish (see Figure 3). However, the block copolymers with a lower balance/symmetry such as *n*-BMA_42_-*b*-GLBT_300_ and all the ZABC with PEHA form a turbid solution (see Figure 3 and Figure 4). Since zwitterionic block-copolymer (GLBT) does not have upper critical solution temperature (UCST), turbidity of the samples is not due to UCST behaviour of the polymer. In addition, we have kept the temperature constant at room temperature. Thus, Turbidity may possibly due to formation of bigger aggregates. The proof for the formation of aggregates will be explained in terms of polydispersity indices of micelles using DLS results in the upcoming section. Previously, we showed that a nearly equal number of chain lengths of hydrophilic and hydrophobic block is the main criteria for bluishness of solution for ZABC with P(*n*-BA). The hydrophobicity of P(*n*-BMA) is higher than that of P(*n*-BA) [22]. Hence, even the block copolymer of ZABC with P(*n*-BMA) with a shorter hydrophobic block forms a bluish solution, i.e., stable micelle formation.

**Figure 5:**
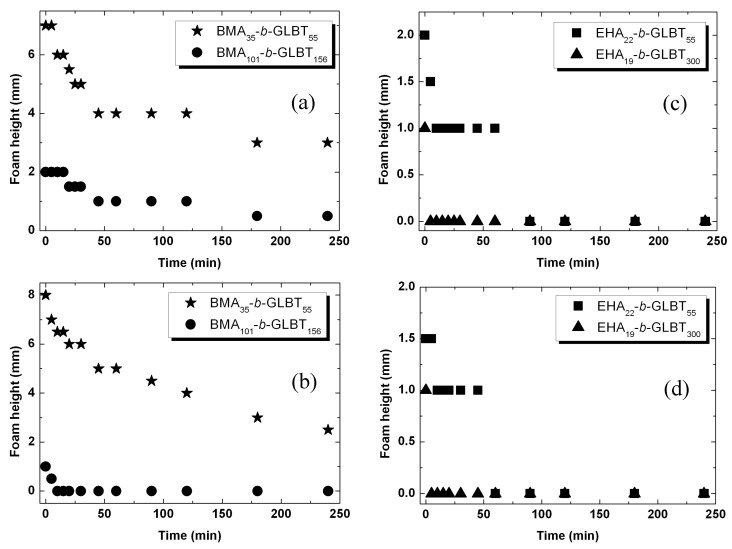
Time-dependent foam height of different ZABC solutions with P(*n*-BMA) and PEHA (concentration = 1 mg/mL): (**a**,**c**) in water; and (**b**,**d**) in 1 M NaCl.

Time-dependent foam heights of the ZABC with P(*n*-BMA) and PEHA are shown in Figure 5. Foam height and stability of ZABC containing P(*n*-BMA) is higher than those of ZABC containing PEHA. For the shortest block length polymers, foam height of former is four times greater than that of latter. ZABC with a shorter chain length have a higher foam height and stability than the ZABC with longer chain lengths. These studies showed that the surface active properties of ZABC depend on the net hydrophobicity of ZABC which is similar to those of IABC [14,15,18].

The present study confirmed that micelle formation and its stability are key factors for non-surface activity also for ZABC in addition to the charged state of ionic block, although an opposite trend to IABC was found for the salt effect on ZABC.

### 3.2. Effect of Salt on CMC

**Figure 6:**
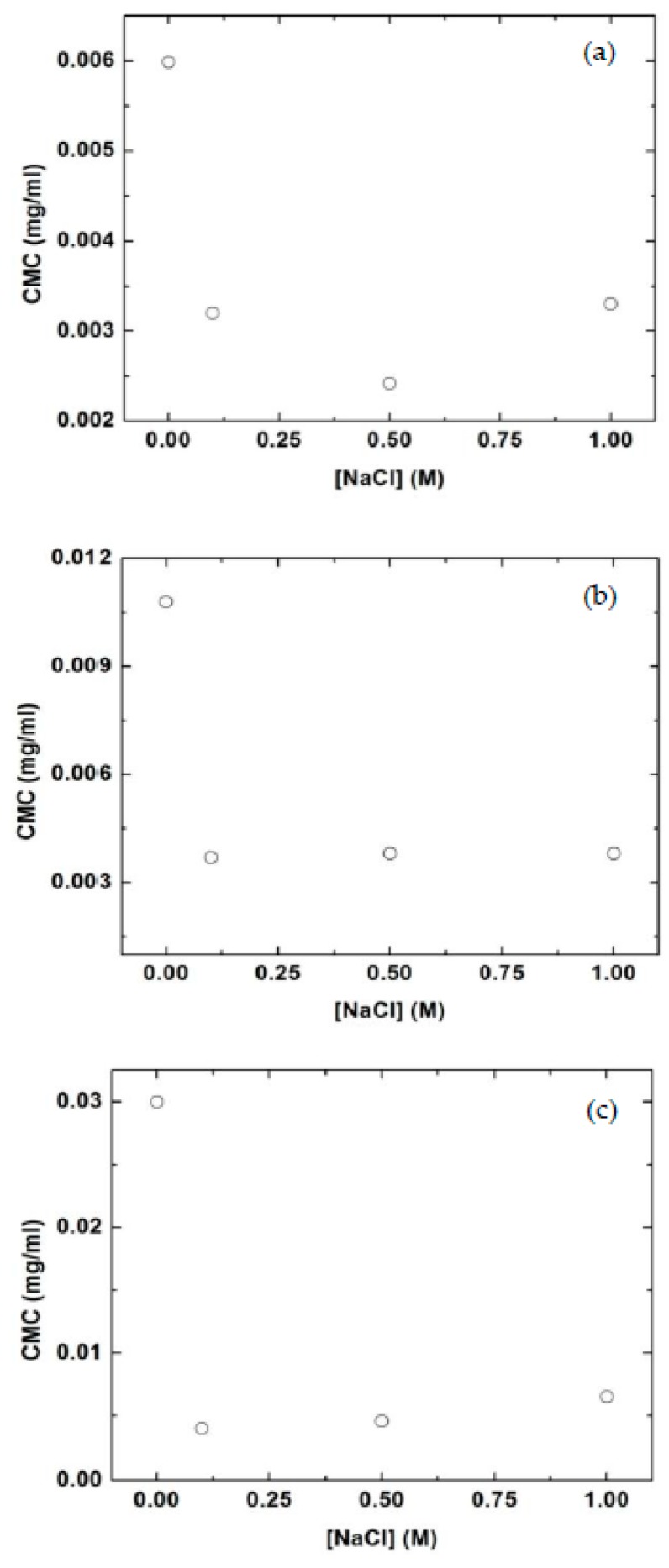
Variation of critical micelle concentration of: (**a**) EHA_22_-*b*-GLBT_55_; (**b**) EHA_15_-*b*-GLBT_117_; and (**c**) EHA_20_-*b*-GLBT_156_ as a function of sodium chloride solutions.

Critical micelle concentrations (CMCs) of ZABC were determined by SLS to elucidate the micellization behaviour of these ZABC. It is difficult to determine CMC of ZABC containing P(*n*-BMA) by SLS unlike PEHA. This may be due to their very low CMC values. Another possible reason may be that the glass transition temperature of P(*n*-BMA) is around room temperature (20 °C) [26]. In contrast, since ZABC with PEHA have a longer hydrophilic chain, CMC was expected to be higher value and hence it was easier to determine their CMC by SLS. Figure 6 shows the salt-dependent CMC of ZABCs. Initially, CMC decreased significantly with the increase in salt concentration up to 0.1 M and then slightly increased with further increase in salt concentration. CMC of low molecular weight ionic surfactants decreased with the increase in salt concentration. Hence, there seems to be a contradiction between the well-known Corrin–Harkins law [37] and the present findings. On the other hand, CMC of non-surface active IABC was found to increase with the increase in salt concentrations [10,16]. Hence, “negative Corrin–Harkins behaviour” is characteristic of non-surface active polymers. ZABCs are slightly surface active without added salt, which can be adsorbed at the water surface and are hard to form micelles, which results in higher CMC. By addition of salt, the situation of zwitterionic group changes as mentioned above and shows a slightly negative charge, which results in appearance of non-surface activity. Hence, the polymer is non-surface active in the presence of salt, CMC increased with increasing added salt concentration. This negative Corrin–Harakins behaviour can be explained as follows: with increasing added salt, the image charge effect at the air–water interface is shielded. Hence, the polymer can easily be adsorbed at the water surface, which makes micelle formation in bulk solution difficult.

### 3.3. Influence of Salt on Hydrodynamic Radius

**Figure 7:**
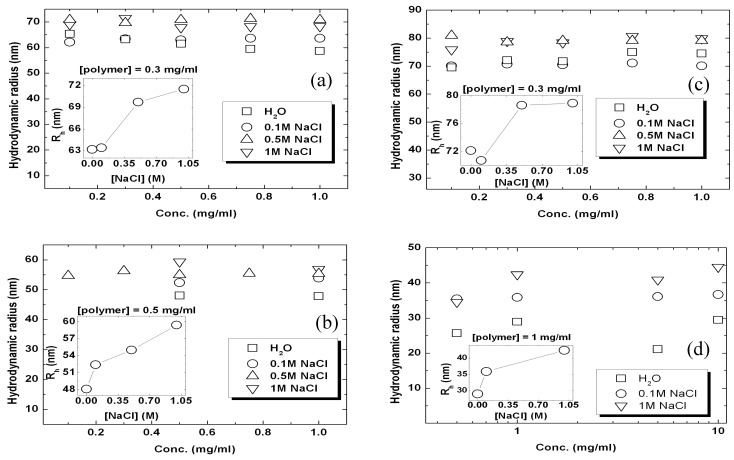
Polymer concentrations dependence of hydrodynamic radius: (**a**) *n*-BMA_62_-*b*-GLBT_117_; (**b**) *n*-BMA_101_-*b*-GLBT_156_; (**c**) *n*-BMA_42_-*b*-GLBT_300_; and (**d**) EHA_20_-*b*-GLBT_156_. Inset shows variation of hydrodynamic radius with different concentrations of sodium chloride.

The hydrodynamic radii of the ZABC micelles having P(*n*-BMA) and PEHA were evaluated by the DLS technique. The roles of hydrophobicity and salt concentrations on the hydrodynamic behaviour of ZABC micelles were examined. Figure 7 shows the polymer concentration dependence of hydrodynamic radius of ZABC micelles having P(*n*-BMA) and PEHA at various salt concentrations. Irrespective of polymer concentration, the hydrodynamic radius of the block copolymer micelle was almost constant. This observation is consistent with our previous observations, although an occasional small increase has been reported [22]. An interesting observation here is the transition from near-monodisperse micelles to polydisperse micelles when the hydrophobic block of ZABC was varied from P(*n*-BMA) to PEHA (Table 1). Polydispersity indices of the micelles can be linked with the turbidity of polymer solutions. ZABC micelle solutions having lower polydispersity indices seem to be transparent (bluish) (Figure 3 and Table 1) while ZABC micelle solutions having higher polydispersity indices were turbid (Figure 4 and Table 1). This observation is probably due to the difference in their hydrophobicity and this will be discussed in the next section. Armes and co-workers [19] observed polydisperse micelles by direct dissolution of the sulphopropylbetaine copolymers in water but near-monodisperse micelles were formed when the preliminary dissolution was carried out in a non-selective solvent (2,2,2-trifluoroethanol). In addition, we observed that at a given polymer concentration the hydrodynamic radius of the ZABC micelle increased with the increase in salt concentration (see inset of Figure 7). This anti-polyelectrolyte effect could be responsible for stretching of betaine block chain by salt addition. Analogous phenomena have been observed for block copolymer containing sulphopropylbetaine [19] and carboxybetaine with P(*n*-BA) [22] after addition of salt.

The dissymmetry ratio is defined as a ratio of light scattering intensities at 45° and 135°. The dissymmetry ratio varied with respect to relative block length of hydrophobic and hydrophilic blocks of ZABC. In the present work, ZABC containing P(*n*-BMA) is more symmetric (almost comparable hydrophobic and hydrophilic block lengths) as compared to ZABC having PEHA (hydrophilic block is longer than hydrophobic block). ZABC with P(*n*-BMA) except *n*-BMA_42_-*b*-GLBT_300_ showed a lower dissymmetry ratio value which is less than 2.1, but ZABC composed of PEHA had a higher dissymmetry ratio of around 3.5 (Table 1). However, the dissymmetry ratio was almost constant even after addition of salt. In addition, the dissymmetry ratio can also be correlated with the turbidity of polymer solutions. For instance, ZABC containing (P(*n*-BMA)) having a lower dissymmetry ratio appeared transparent except *n*-BMA_42_-*b*-GLBT_300_ (Figure 3 and Table 1), whereas ZABC having a higher dissymmetry ratio (PEHA) appeared turbid (Figure 4 and Table 1). DLS studies revealed that the micellization properties of ZABC consisting of P(*n*-BMA) seems to be different from those of ZABC having PEHA.

### 3.4. Hydrophobicity, Salt and Block Length-Dependent Aggregation Number and Second Virial Coefficient of Micelles

We analysed the SLS data using Zimm plots (Figure S1) as described previously [22]. Zimm plots are summarized in Table 2. First, the effects of the hydrophobic and hydrophilic block lengths on the aggregation numbers of ZABC were examined. The aggregation number (*N*_agg_) was inversely proportional to the chain length of polymers. For example, in the case of ZABC with P(*n*-BMA), when the block length of P(*n*-BMA) was changed from 35 to 101 units and that of PGLBT was increased from 55 to 156 units, the aggregation number (*N*_agg_) decreased from 555 to 117, by five times. We found recently that, for ZABC with P(*n*-BA), *N*_agg_ decreased by five times for similar variation of soluble block length [22]. The present findings for ZABC having P(*n*-BMA) were analogous to our recent results for ZABC with P(*n*-BA) [22]. However, the *N*_agg_ of ZABC having PEHA was reduced 10 times (from 891 to 91) when the chain length of the PGLBT block varied from 55 to 156 units and that of PEHA was almost constant. The difference in decrease of *N*_agg_ with change in hydrophobic block (from P(*n*-BMA) to PEHA) could be due to higher hydrophobic nature of PEHA chain. On the other hand, Khougaz et al. [38] found that the aggregation number of IABC was affected by the length of the hydrophobic block more than the hydrophilic block. In addition, Armes et al. [19] found that for the block copolymer having sulphopropylbetaine, the value of *N*_agg_ increased more with the increase in the length of the hydrophobic block rather than with the decrease in the hydrophilic block.

Next, we noticed that *N*_agg_ decreased with addition of salt. In the P(*n*-BMA)-*b*-PGLBT systems, salt addition decreased *N*_agg_ from 555 to 146 in *n*-BMA_35_-*b*-GLBT_55_ and from 117 to 32 in BMA_101_-*b*-GLBT_156_. We have observed similar phenomena for ZABC composed of PEHA where *N*_agg_ was about 6 times lower (Table 2). Our observations are consistent with earlier studies on block copolymers having sulphopropylbetaine [19] and carboxybetaine [22]. In contrast, Khougaz et al. [38] showed that the aggregation numbers of IABC initially increased with increasing salt concentration and then reached saturation. From the present study, it is clear that the increase in chain length of polymer affects the value of *N*_agg_ slightly higher margin as compared to salt.

Studies on the effect of hydrophobicity and salt on the second virial coefficient (*A*_2_) showed that the value of *A*_2_ changed from positive to negative when the hydrophobic block of ZABC is changed from P(*n*-BMA) to PEHA (Table 2). This indicates that the solubility of ZABC having P(*n*-BMA) in water could be higher than that of ZABC having PEHA in water. Similar to *N*_agg_, the value of *A*_2_ also decreased with increase in block length of ZABC. For example, the values of *A*_2_ decreased with the increase in both soluble and insoluble blocks of ZABC containing P(*n*-BMA) and values were 4.7 × 10^−5^ and 2.7 × 10^−5^ cm^3^·mol·g^−2^ for *n*-BMA_35_-*b*-GLBT_55_ and *n*-BMA_101_-*b*-GLBT_156_, respectively, in water. In addition, the values of *A*_2_ decreased with the increase in salt concentration. In the presence of 1 M NaCl, the values of *A*_2_of *n*-BMA_35_-*b*-GLBT_55_ and *n*-BMA_101_-*b*-GLBT_156_ decreased to 0.25 × 10^−5^ and 1 × 10^−5^ cm^3^·mol·g^−2^, respectively. Analogous phenomenon has been observed for ZABC having PEHA (see Table 2). The decrease in polymer-solvent interaction is responsible for the decrease in value of *A*_2_ with increase in block length and NaCl concentration. These results are consistent with the decrease in CMC with increase in salt concentration. Khougaz and co-workers [38] reported that in IABC, the values of *A*_2_ for fixed hydrophobic block length PS(23)were increased from −3.5 × 10^−4^ to −0.36 × 10^−4^ cm^3^·mol·g^−2^ with the increase in chain length of hydrophilic block (PANa) from 44 to 300. This behaviour implies that IABC with a longer PANa chain interacts with the solvent in a more favourable manner than IABC with a shorter PANa chain. The solubility of the block copolymer was expected to increase with the increase in the soluble block length. However, the values of the radius of gyration (*R*_g_) were hardly affected by the increase in the chain length and salt concentration (Table 2).

To determine the value of core radius (*R_c_*) of micelles, we substituted the known values of *M*_w_ and *N*_agg_ (from Zimm plots) in the equation $R_{c}=\sqrt[{3}]{\frac{3N_{agg}N_{}M_{w}}{4\pi \rho N_{A}}}$, where *N* is the block length of the hydrophobic chain, *M*_w_ is the molecular weight of the hydrophobic monomer, ρ is the density of the bulk polymer (for P(*n*-BMA) and PEHA approximately 1.0 mg/mL and 0.9 mg/mL respectively) and *N*_A_ is the Avogadro’s number. We estimated the packing parameter (β) of micelle using the equation $\beta =\frac{V_{H}}{L_{C}A_{0}}$, where *V_H_* is the volume occupied by the hydrophobic chain, *L*_c_ is the counter length of the hydrophobic chain (≈core radius, *R*_c_) and *A*_0_ is the surface area of the hydrophilic chain. The morphology of polymeric aggregates was identified from the values of *β* [39,40]. From the values of *V*_H_ (=4π*R*_c_^3^/3*N*_agg_), *L*_c_ (=*R*_c_) and $A_{0}=\left(4\pi {\left(\left(R_{h}+R_{c}\right)/2\right)}^{2}\right)/N_{agg}$, packing parameters were calculated and summarized in Table 2. Irrespective of the polymer, the values of *β* for the ZABC micelles were less than 0.3 and this implies that micelles formed from the block copolymers are spherical even after addition of salt. The surface area of the hydrophilic chain increased after addition of salt and hence the packing parameter tended to decrease below 0.3. For instance, the surface area of hydrophilic chain of *n*-BMA_35_-*b*-GLBT_55_ increased from 74 to 267 nm^2^ when the concentration of NaCl increased from 0 M NaCl to 1 M NaCl. This phenomenon enables ZABC micelles to retain their spherical shape after salt addition. We observed similar behaviour for ZABC micelles having P(*n*-BA) [22]. The results obtained from the Zimm plots are consistent with the hydrodynamic behaviour of ZABC micelles. However, our preceding study on the micellization behaviours of IABC with strong acid groups, poly(hydrogenated isoprene)-*b*-poly(styrene sulphonate), showed transition from sphere to rod after addition of salt [11]. The surface area of the hydrophilic chain and corona thickness of IABC micelles decreased after addition of salt and hence the packing parameter might be above 0.3. Thus, the micelles formed from IABC may undergo transition from sphere to rod.

**Figure 8:**
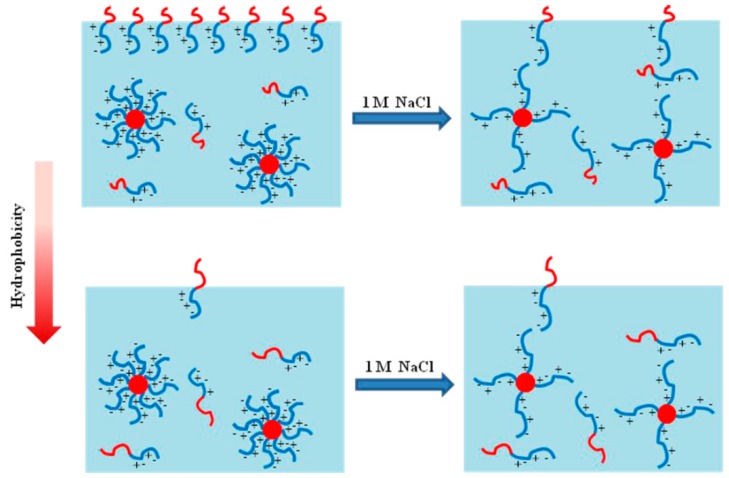
Schematic illustration of influence of hydrophobicity and salt on the air–water interfacial and micellization properties of ZABCs having P(*n*-BMA) and PEHA. Blue and red curves respectively indicate zwitterionic and hydrophobic polymers. Hydrophobic core of the micelles are represented by red circles or balls.

Figure 8 illustrates the influence of hydrophobicity and salt on the surface activity and micellization behaviour of ZABC. Increase in the hydrophobicity of ZABC by changing the hydrophobic block (from P(*n*-BMA) to PEHA), caused transition from surface active to non-surface active. Similarly, addition of salt caused transition of the ZABC composed of P(*n*-BMA) (particularly for *n*-BMA_42_-*b*-GLBT_300_) from surface active to non-surface active polymers. Further salt addition increased the hydrodynamic radius of the micelle and decreased the aggregation number of the micelle.

## 4. Conclusions

Various ZABCs with P(*n*-BMA) or PEHA as a hydrophobic block and carboxybetaine as a hydrophilic block were successfully synthesized by the RAFT polymerization method. The two parameters, hydrophobicity and salt, were varied separately to examine their role on the surface activity and micellization behaviour of ZABC. Surface tension measurements and foam formation observations revealed that the hydrophobicity of hydrophobic block present in ZABC is the predominant factor for the surface active and foam forming behaviour of ZABC. When the hydrophobicity of the hydrophobic block of ZABC was increased by introducing PEHA instead of P(*n*-BMA), ZABC showed transition from surface active to non-surface active. Similar observations were observed for IABC [14]. A possible reason for this behaviour could be that ZABC with higher hydrophobicity can form micelles, which is more stable than the adsorbed state at the air–water interface. Salt addition caused transition of ZABC with P(*n*-BMA) from surface active to non-surface active. This may be due to zwitterionic to anionic transition of the betaine block. The value of CMC of ZABC slightly increased after addition of salt, which is typical for non-surface active polymers. The increase in the value of hydrodynamic radius with increase in salt concentration at a given polymer concentration might be due to the anti-polyelectrolyte effect, i.e., increase in chain length of hydrophilic corona. This observation is supported by the increase in surface area of hydrophilic betaine corona after the addition of salt. However, the aggregation number and second virial coefficient of the micelles tended to decrease with the increase in chain length, and with the addition of salt. DLS and Zimm plot results revealed that ZABC containing P(*n*-BMA) could form more monodisperse micelles than those containing PEHA. When the hydrophobic block P(*n*-BMA) was replaced by PEHA in ZABC having a fixed zwitterionic hydrophilic block, the value of *A*_2_ changed from positive to negative. This is due to the decrease in the solubility of the polymer. Unlike IABC micelles, the micellar shape of ZABC micelles was not affected by the addition of salt, which was confirmed from the packing parameter values of block copolymer micelles (less than 0.3). The present study revealed that ZABC becomes non-surface active when the betaine block is changed to an ionic state by addition of salt or when very stable micelles are formed in bulk solutions with high enough hydrophobicity.

## Figures and Tables

**Table 1:** Effect of hydrophobicity and salt on dissymmetry ratio and polydispersity indices of the micelles.

Diblock Copolymer	*Z*_d_^a^		PDI ^b^	
	Water	1 MNaCl	Water	1 MNaCl
*n*-BMA_35_-*b*-GLBT_55_	1.6	1.5	0.16	0.13
*n*-BMA_62_-*b*-GLBT_117_	2.1	2	0.13	0.13
*n*-BMA_101_-*b*-GLBT_156_	1.5	1.5	0.11	0.08
*n*-BMA_42_-*b*-GLBT_300_	2.7	2.6	0.2	0.19
EHA_22_-*b*-GLBT_55_	3.5	3.2	0.29	0.28
EHA_15_-*b*-GLBT_117_	4.4	4.5	0.29	0.29
EHA_20_-*b*-GLBT_156_	3.9	3.9	0.3	0.32
EHA_19_-*b*-GLBT_300_	3.5	3.7	0.19	0.21

^a^ Dissymmetry ratio *Z_d_* = *I*_45_/*I*_135_; *I*_45_ and *I*_135_ are scattering intensities at angles 45° and 135° respectively. ^b^ PDI: Polydispersity indices of the micelles.

**Table 2:** Micellar properties of ZABC micelles.

Diblock Copolymer	*N*_agg_^a^		*A*_2_ × 10^5^ (cm^3^·mol·g^−2^) ^b^		*R*_g_ (nm) ^c^		*β* ^d^	
	Water	1 MNaCl	Water	1 MNaCl	Water	1 MNaCl	Water	1 MNaCl
*n*-BMA_35_-*b*-GLBT_55_	555	146	4.7	0.25	65.9	58.1	0.031	0.02
*n*-BMA_101_-*b*-GLBT_156_	117	32	2.7	1.0	58.8	55.8	0.032	0.01
EHA_22_-*b*-GLBT_55_	891	152	−3.5	−17.6	203.3	172.8	0.1	0.03
EHA_20_-*b*-GLBT_156_	91	13	−3.1	−88.1	218.7	139.8	0.032	0.01

^a^ *N*_agg_: Aggregation number. ^b^ *A*_2_: Second virial coefficient. ^c^ *R*_g_: Radius of gyration. ^d^ *β*: Packing parameter.

## Supplementary Materials

Supplementary Materials are available online at www.mdpi.com/2073-4360/9/9/412/s1.
