# Supplementary material for: Surface Active to Non-Surface Active Transition and Micellization Behaviour of Zwitterionic Amphiphilic Diblock Copolymers: Hydrophobicity and Salt Dependency
Source: Polymers (Basel). 2017 Sep 5;9(9):412. doi: 10.3390/polym9090412 (PMC6418639; doi:10.3390/polym9090412)
Supplement: Supplementary file 1 [file polymers-09-00412-s001.pdf]

Supplementary Materials

Surface active to non-surface active transition and micellization behaviour of zwitterionic amphiphilic diblock copolymers: hydrophobicity and salt dependency

Sivanantham Murugaboopathy and Hideki Matsuoka

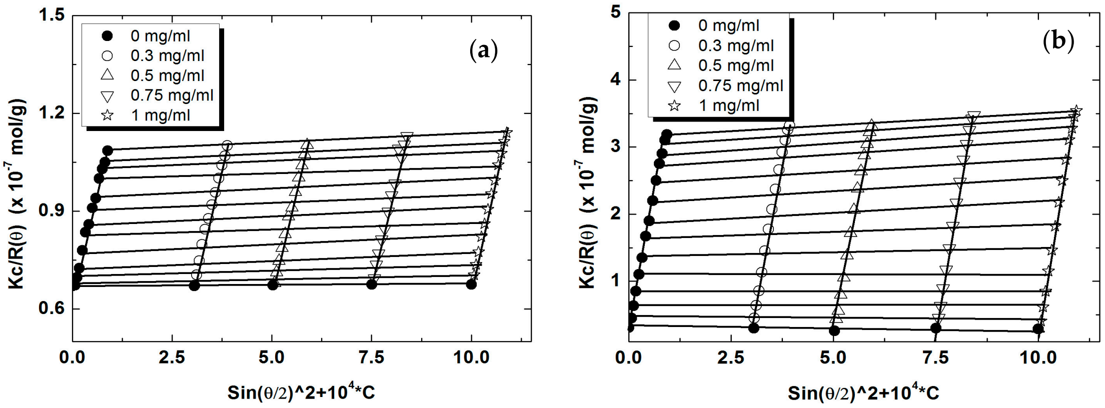

Figure S1. Typical Zimm plots for ZABC having P(*n*-BMA) and PEHA. (a) *n*-BMA_{101}-*b*-$GLBT_{156}$ in 1M NaCl and (b) EHA_{20}-*b*-$GLBT_{156}$ in water.

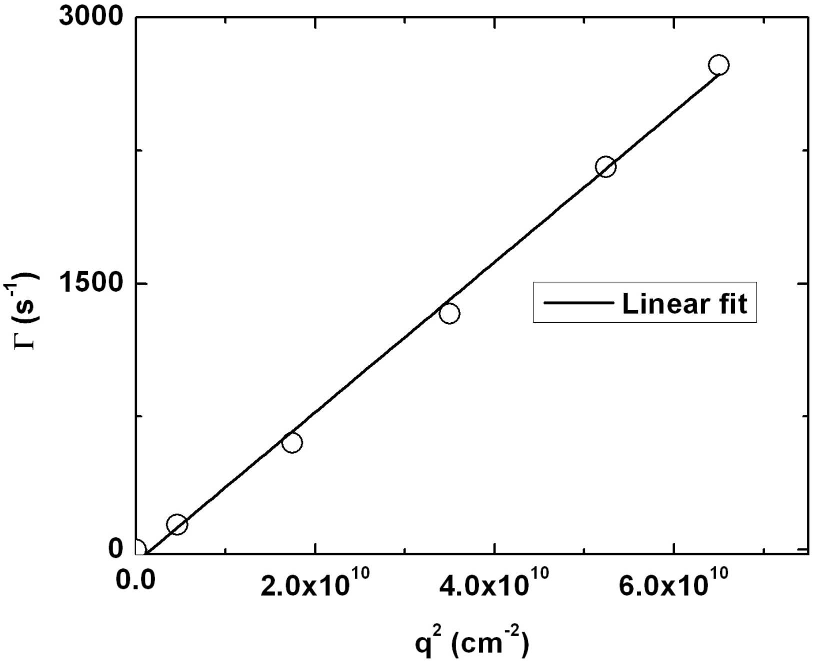

Figure S2. Plots between Γ vs. q^2 for *n*-BMA_{35}-*b*-$GLBT_{55}$ in water.

Table S1. Polymerization conditions for the synthesis of homopolymers(PGLBT) and block copolymers (P(*n*-BMA)-*b*-PGLBT) and (PEHA-*b*-PGLBT) (70 °C).

Polymer	Monomer (mmol)	RAFT Agent (mmol) [a]	Initiator (mmol) [b]	Water (mL)	DMF (mL)	Methanol (mL)	Polymerization Time (h)
PGLBT – 1	18.6	0.2	0.1	6	1.5		2
PGLBT – 2	37.2	0.2	0.1	6	1.5		1.5
PGLBT – 3	37.2	0.19	0.097	6	1.5		2
PGLBT – 4	37.2	0.099	0.1	6	1.5		2
n-BMA$_m$-*b*-GLBT$_{n'}$-1	6.4	0.064	0.52			5	24
n-BMA$_m$-*b*-GLBT$_{n'}$-2	6.4	0.074	0.35			5	24
n-BMA$_m$-*b*-GLBT$_{n'}$-3	3.2	0.032	0.26			5	24
n-BMA$_m$-*b*-GLBT$_{n'}$-4	4.8	0.032	0.26			5	24
EHA$_m$-*b*-GLBT$_{n'}$-1	2.4	0.097	0.39			5	24
EHA$_m$-*b*-GLBT$_{n'}$-2	0.6	0.024	0.2			5	24
EHA$_m$-*b*-GLBT$_{n'}$-3	0.6	0.024	0.2			5	24
EHA$_m$-*b*-GLBT$_{n'}$-4	2.4	0.014	0.43			5	24

[a] For synthesis of diblock copolymers, amount of macro RAFT agent in mmol. [b] for synthesis of homopolymers and diblock copolymers, 4,4′-Azocyanovaleric acid (ACVA) and 2,2′-azobisisobutyronitrile (AIBN) were respectively used as initiator. DMF: dimethylformamide. GLBT: carboxybetaine. *n*-BMA: *n*-butylmethacrylate. EHA: 2-ethylhexylacrylate.

Table S2. Molecular characteristics of homopolymers(PGLBT) and diblock copolymers (P(*n*-BMA)-*b*-PGLBT) and (PEHA-*b*-PGLBT).

Polymer	M_n [a] (g/mol)	PDI [b]	Degree of polymerization	
			m	*n′*
PGLBT – 1	11900	1.13		55
PGLBT – 2	25200	1.2		117
PGLBT – 3	33600	1.15		156
PGLBT – 4	64500	1.16		300
n-BMA$_m$-*b*-GLBT$_{n'}$-1			35	55
n-BMA$_m$-*b*-GLBT$_{n'}$-2			62	117
n-BMA$_m$-*b*-GLBT$_{n'}$-3			101	156
n-BMA$_m$-*b*-GLBT$_{n'}$-4			42	300
EHA$_m$-*b*-GLBT$_{n'}$-1			22	55
EHA$_m$-*b*-GLBT$_{n'}$-2			15	117
EHA$_m$-*b*-GLBT$_{n'}$-3			20	156
EHA$_m$-*b*-GLBT$_{n'}$-4			19	300

[a] M_n: Number averaged molecular weight (determined by GPC). [b] PDI: Polydispersity index (M_w/M_n) (determined by GPC). *m*, *n′*: The degree of polymerization of hydrophobic and hydrophilic blocks respectively(*m* was determined by NMR). GLBT: carboxybetaine; *n*-BMA: *n*-butylmethacrylate. EHA: 2-ethylhexylacrylate.
